# Phenotypic Resistance of *Campylobacter* Isolates to an Extended Panel of Antibiotics

**DOI:** 10.4269/ajtmh.26-0268

**Published:** 2026-07-30

**Authors:** Lucero Romaina Cachique, Francesca Schiaffino, Maribel Paredes Olortegui, Katia Manzanares, Tackeshy Pinedo Vasquez, Alejandra Castro, Juana Najarro, Kerry K. Cooper, Evangelos Mourkas, Ben Pascoe, Pablo Peñataro Yori, Craig T. Parker, Margaret N. Kosek

**Affiliations:** ^1^ Asociacion Benefica PRISMA, Iquitos, Peru;; ^2^ Faculty of Veterinary Medicine, Universidad Peruana Cayetano Heredia, San Martin de Porres, Lima, Peru;; ^3^ Division of Infectious Diseases and International Health, School of Medicine, University of Virginia, Charlottesville, Virginia, USA;; ^4^ School of Animal and Comparative Biomedical Sciences, University of Arizona, Tucson, Arizona, USA;; ^5^ Zoonosis Science Center, Department of Medical Sciences, Uppsala University, Uppsala, Sweden;; ^6^ Ineos Oxford Institute for Antimicrobial Research, Department of Biology, University of Oxford, Oxford, United Kingdom

## Abstract

Because of the global increase in resistance of *Campylobacter* to both fluoroquinolones and macrolides, we explored phenotypic resistance using traditional Kirby–Bauer methods in human- and animal-derived *Campylobacter* isolates that were concurrently resistant to both azithromycin and ciprofloxacin to an expanded panel of antimicrobials, including clindamycin, fosfomycin, ampicillin and sulbactam, amoxicillin and clavulanic, fosfomycin, tigecycline, and imipenem. Of 235 *Campylobacter* isolates resistant to ciprofloxacin and azithromycin, more than 94.8% of *Campylobacter jejuni* and *Campylobacter coli* were resistant to clindamycin, 68.5% were resistant to ampicillin and sulbactam, 2.6% were resistant to amoxicillin and clavulanic, and 36.2% were resistant to fosfomycin. Less than 2% of isolates were resistant to tigecycline, and there was no observed resistance to imipenem. The results suggest that amoxicillin and clavulanic warrant further evaluation for use in *Campylobacter* enteritis in ambulatory patients, but it points to no clear alternative oral therapies. Study findings were reassuring in demonstrating continued universal susceptibility to carbapenems.

## INTRODUCTION

*Campylobacter* infections are a leading global cause of bacterial enteritis.^1^^–^^3^ Additionally, *Campylobacter* are among the foremost pathogens associated with foodborne illnesses, traveler’s diarrhea, and animal contact in high-income countries, causing 1.9 million illnesses and up to 13,000 hospitalizations in the United States.^4^^–^^8^ Although antimicrobials are not advised for treatment of uncomplicated gastroenteritis, antimicrobial therapy is advised for patients with severe symptoms, dysentery, and persistent infections as well as in patients who are immunocompromised or pregnant.^9^

Fluoroquinolones (ciprofloxacin [CIP]) and macrolides (azithromycin [AZM]) are the two most effective oral antimicrobial treatments for campylobacteriosis in sensitive strains, and they have been shown to shorten the duration of illness in clinical trials.^10^ *Campylobacter* strains demonstrating resistance to CIP have shown a steady increasing trend from 2013 to 2024 in the United States, with 23.2% of isolates resistant in 2013 and 41.1% of isolates resistant in 2024 according to the National Antimicrobial Resistance Monitoring System (NARMS).^11^ Macrolide resistance in *Campylobacter jejuni* remained stable in the United States between 2013 and 2019; however, between 2000 and 2024, it rose but remains relatively low, with only 2.7% of isolates demonstrating resistance to azithromyicn.^12^^,^^13^ The occurrence of *C. jejuni* and amoxicillin and clavulanic *Campylobacter coli* isolates resistant to macrolides has further been evidenced in other scenarios, such as the outbreak in the United States linked to puppies, making it clear that there is a need to evaluate additional antibiotics to be prepared for similar future outbreaks and progressive antimicrobial resistance (AMR) in campylobacteriosis.^14^^–^^16^ This study attempted to define possible alternative therapies among strains resistant to both CIP and AZM.

The increase in AMR among *C. jejuni* and *C. coli* has occurred globally and is likely the result of the extensive use of antimicrobials in livestock animals, especially in regions with poor or minimal enforcement of restrictions in the use in agroindustry. In Peru, prevalence trends of AMR in *C. jejuni* and *C. coli* have primarily focused on resistance to CIP and AZM in humans given their importance in the treatment of human illnesses.^17^^–^^19^ Resistance to CIP has increased dramatically in the Peru from 2001 to 2010^17^^,^^18^ and remained at higher levels through 2022.^19^^,^^20^ Genomic analysis of *C. jejuni* and *C. coli* isolates in Iquitos, Peru has identified specific chromosomal mutations for resistance, including a *gyrA* Thr86Ile mutation associated with fluoroquinolone resistance, the 23S ribosomal RNA (rRNA) mutations associated with macrolide resistance, and resistance enhancing mutations in the cmeABC operon (RE-cmeABC) conferring multidrug resistance (MDR; (defined as nonsusceptibility to at least one antibiotic from three or more antimicrobial classes) to the CmeABC efflux pump.^20^^–^^25^

Given the lack of clearly defined additional options to treat clinical cases of campylobacteriosis demonstrating resistance to both CIP and AZM, there is a need to evaluate additional antibiotics that are commonly used to treat gastrointestinal infections, particularly oral therapies. We explored in vitro phenotypic resistance patterns in a broad collection of *C. jejuni* and *C. coli* isolates derived from human and animal fecal samples to an expanded set of antibiotics that have not been widely evaluated.

## MATERIALS AND METHODS

Infant fecal samples, animal fecal samples, and meat were assessed during a period of an ongoing cohort study conducted in the Peruvian Amazon from 2021 to 2025. Human fecal samples were collected monthly or every time that the child had diarrhea. Animal fecal samples were derived from a nested case–control study associated with the same cohort or from concurrent surveillance conducted throughout the duration of the cohort study; livestock (chickens, pigs, and cows), household animals, and chicken meat were assessed for the presence of *Campylobacter*. The study was approved by the Institutional Review Boards of Asociacion Benefica PRISMA (CE0035.26) and the University of Virginia (HSR210091) and the Institutional Committee on the Ethical Use of Animals of Universidad Peruana Cayetano Heredia (21001). Written informed consent to participate in the study was obtained from the parents or legal guardians of children. Participants consented for further use of biological specimens.

Meat samples were placed in 50 mL of 0.01% peptone water and manually homogenized for 3 minutes. The sample liquid was placed in a 50 mL conical tube and centrifuged at 5,000 revolutions per minute for 15 minutes. The pellet was resuspended in 10 mL of 0.01% peptone water, and a 1:10 dilution was further performed; 300 *µ*L of the sample was placed in Bolton Broth (Oxoid, Thermo Fisher Scientific, Waltham, MA) and placed in microaerophilic conditions at 37°C for 4 hours and at 42°C for 12 hours. A volume of 100 *µ*L of the sample was then inoculated using the same method described for fecal samples.

Human and animal fecal samples were cultured using Columbia Blood Agar Base (Oxoid, Thermo Fisher Scientific) supplemented with 5% lysed horse blood and an S-pack filter of 0.45 *µ*M and 47 mm in diameter (Merck Millipore, Burlington, MA) in microaerophilic conditions (1% O_2_ + 10% CO_2_ + 10% H_2_ + balance N_2_) at 37°C. Colonies compatible with *Campylobacter* morphology were further confirmed as *Campylobacter* spp. or *C. jejuni*/*C. coli* using a duplex quantitative polymerase chain reaction targeting the 16S rRNA and an *rpsS*/*rpsK* gene in *Campylobacter*.^26^^,^^27^ Only isolates for which a species (*C. jejuni* or *C. coli*) could be assigned were included in the final dataset for analysis.

Phenotypic antimicrobial susceptibility testing (AST) was performed using standard disk diffusion testing and Mueller–Hinton agar with 5% mechanically defibrinated horse blood.^19^ A direct colony suspension was made to 0.5 McFarland standard, and resistance to the following antibiotics was tested at 42°C for 24 hours in a microaerobic atmosphere of 10% CO_2_, 5% O_2_, and 85% N_2_ per the Clinical & Laboratory Standards Institute (CLSI) M45 protocol: CIP (5 *µ*g), erythromycin (ERY; 15 *µ*g), AZM (15 *µ*g), tetracycline (TET; 30 *µ*g), gentamicin (GEN; 10 *µ*g), amoxicillin and clavulanic acid (AMC; 20 and 10 *µ*g, respectively), chloramphenicol (CHL; 30 *µ*g), imipenem (IMP; 10 *µ*g), clindamycin (CLI; 2 *µ*g), fosfomycin (FOSF; 200 *µ*g), ampicillin and sulbactam (SAM; 10 and 10 *µ*g, respectively), and tigecycline (TGC; 15 *µ*g). An epsilometer test (E test) for AZM was additionally prepared for all isolates. This panel of antibiotics tested exceeds the spectrum of the NARMS. Zone diameter break points (millimeters) for *Campylobacter* spp. from the CLSI M45 were applied to assess CIP, ERY, AZM, and TET resistance. The CLSI zone diameter break points (millimeters) for *Enterobacteriaceae* were used for GEN, AMC, AMP, CHL and IMP, CLI, and FOSF and SAM given that there are no established break points for *Campylobacter* (Supplemental Table 1).

## RESULTS

A total of 235 *Campylobacter* isolates from 228 fecal samples were concurrently resistant to AZM and CIP. Of these, 111 animal-derived isolates and 67 human-derived isolates were determined to be *C. coli.* Additionally, 14 human-derived isolates and 43 animal-derived isolates were determined to be *C. jejuni.* A single human sample and six chicken-derived samples were determined to have a coinfection of *C. jejuni* and *C. coli*, and thus, both isolates were included in the analysis*.* This higher prevalence of *C. coli* relative to *C. jejuni* is because of the selection criteria for CIP and AZM resistance; in general, 80% of isolates from pediatric populations and chickens in this area are *C. jejuni*, and only 20% are *C coli.* The prevalences of resistance to each tested antibiotic are shown separately for animal and human samples as well as *C. jejuni* and *C. coli* isolates in Table 1. Briefly, 169 of 178 *C. coli* and 54 of 57 *C. jejuni* had E-test results available to report MIC. All available isolates showed resistance to AZM (*n* = 8 with minimal inhibitory concentration [MIC] of between >16 and 75 *µ*g/mL; *n* = 215 with MIC of >256 *µ*g/mL), all of which would be considered resistant. Resistance to antibiotics included in the extended panel (CLI, FOSF, AMPSUL, and TGC) varied. Antimicrobial resistance patterns of both *C. jejuni* and *C. coli* in humans and isolates attained from animals were largely similar, but *C. coli* consistently demonstrated higher levels of prevalence of AMR to the antibiotics tested. Combining results for *C. jejuni* and *C. coli* among human and animal isolates, 94.8% of isolates were resistant to CLI, 68.5% were resistant to amoxicillin and sulbactam, 36.2% were resistant to FOSF, and only 2.60% were resistant to amoxicillin and clavulanic. For human isolates only, resistance to CLI was 97.5%, resistance to AMPSUL was 81.5%, there was no resistance to amoxicillin and clavulanic, and resistance to FOSF was 42.0%.

**Table 1:** The distribution of azithromycin-resistant and ciprofloxacin-resistant *Campylobacter jejuni* and *Campylobacter coli* strains derived from human and animal isolates

Antibiotic	Human Isolates (*n* = 81), %						Animal Isolates (*n* = 154), %					
	*Campylobacter jejuni* (*n* = 14)			*Campylobacter coli* (*n* = 67)			*Campylobacter jejuni* (*n* = 43)			*Campylobacter coli* (*n* = 111)		
	R	I	S	R	I	S	R	I	S	R	I	S
Erythromycin (15 *µ*g)	100.0	0.0	0.0	98.5	0.0	1.5	100.0	0.0	0.0	100.0	0.0	0.0
Tetracycline (30 *µ*g)	92.9	0.0	7.1	95.5	1.5	3.0	100.0	0.0	0.0	92.7	0.0	7.2
Amoxicillin and clavulanic acid (20 and 10 *µ*g, respectively)	0.0	0.0	100.0	0.0	4.5	95.5	0.0	2.3	97.7	5.4	10.8	83.8
Gentamicin (10 *µ*g)	35.7	0.0	64.3	70.1	0.0	29.9	60.5	0.0	39.5	48.6	0.9	50.5
Chloramphenicol (30 *µ*g)	0.0	0.0	100.0	7.6	1.5	90.9	4.7	2.3	93.0	7.2	4.5	88.2
Imipenem (10 *µ*g)	0.0	0.0	100.0	0.0	0.0	100.0	0.0	0.0	100.0	0.0	0.0	100.0
Clindamycin (2 *µ*g)	85.7	0.0	14.3	100.0	0.0	0.0	97.7	0.0	4.7	91.8	0.0	8.2
Ampicillin and sulbactam (10 and 10 *µ*g, respectively)	64.3	14.3	21.4	85.1	1.5	13.4	69.8	9.3	20.9	58.7	4.6	36.7
Fosfomycin (200 *µ*g)	28.6	7.1	64.3	44.8	3.0	52.2	30.2	4.7	65.1	34.3	0.0	65.7
Tigecycline (15 *µ*g)	0.0	0.0	100.0	1.5	0.0	98.5	2.4	0.0	97.6	0.9	0.0	99.1

I = intermediate; R = resistant; S = sensitive.

## DISCUSSION

Culture-independent diagnostic tests are increasingly used for the diagnosis of *Campylobacter*. Given the increasing prevalence of drug resistance among isolates, the culture of positive samples for antimicrobial susceptibility testing should be strongly considered by clinicians, particularly among returning travelers who have been shown to have a higher prevalence of resistance to both CIP and ERY.^28^ In this study, we focused on a set of isolates with demonstrated resistance to CIP and AZM to determine what other practical treatment options exist. The expanded set of antibiotics tested was used to evaluate alternative antimicrobials, particularly oral regimes, for the most common cause of bacterial gastroenteritis. Prior studies of CIP-resistant strains identified FOSF, AMC, and CHL as possible alternatives.^29^ Fosfomycin has been used for the treatment of infectious gastroenteritis for other MDR causes of enterocolitis, including *Shigella sonnei*,^30^ including in clinical trials of *Campylobacter* gastroenteritis with favorable outcomes.^31^ Crossresistance to other antimicrobials is nominal, and it would theoretically be an option for treating drug-resistant dysentery in contexts where rapid and complete phonotypic and genotypic testing may not be rapidly available. Unfortunately, human isolates revealed too high a prevalence of resistance to make this a feasible strategy in this setting. Clindamycin is less appealing as it fails to simultaneously treat other principal causes of bacterial enteritis and was also shown to have a high prevalence of nonsusceptibility. Amoxicillin and clavulanic have consistently been identified in in vitro testing of resistant clinical isolates of *Campylobacter* as a feasible oral option.^32^^,^^33^ The discrepancy of in vitro results between AMC and AMPSUL is notable and likely the result of the β-lactamase enzyme *bla*_OXA-61_’s relative resistance to sulbactam as compared with clavulanic acid.^34^ Amoxicillin and clavulanic acid was the only oral antimicrobial agent that had >80% of isolates demonstrating susceptibility, which would be a conservative threshold of AST to maintain an antibiotic for empiric treatment.^35^ However, even with these in vitro results, clinical trials would be necessary to demonstrate efficacy before recommending this therapy as there are numerous results in the literature for enteric diseases in which agents that demonstrated in vitro susceptibility were found to have significant rates of failure in the treatment of clinical disease.^36^^,^^37^ Furthermore, there have been two cases of clearly described treatment failure^38^ in patients with isolates that displayed in vitro susceptibility.

A weakness of the present study is the reliance on disk diffusion testing for phenotypic AMR testing instead of MICs. This was done by well-standardized methods for simplicity and low cost. Although MIC testing is preferable, the discordance between the two methods is in general limited in *Campylobacter*^39^ to key antibiotics and extremely unlikely to be different to the degree that would change the interpretation of high levels of resistance that preclude the use of CLI and FOSF for *Campylobacter* in this region.

## CONCLUSION

Fortunately, multidrug-resistant *Campylobacter* has typically been sensitive to carbapenems, with nonsusceptibility rates of <1% consistent with our finding of no nonsusceptibility in this study.^16^ We tested TGC as an alternative agent for highly resistant Gram-negative infections, and we also found nearly universal susceptibility (<2%). Although these findings further clarify options for patients with life-threatening illnesses, oral options require further evaluation for the management of patients with moderate to severe diarrhea in populations in endemic high-burden settings and for large outbreaks that make it challenging to rely solely on injectable agents.

## Supplemental Materials

10.4269/ajtmh.26-0268 Supplemental Materials
