# Supplementary material for: Phenotypic Resistance of Campylobacter Isolates to an Extended Panel of Antibiotics
Source: Am J Trop Med Hyg. 2026 Jul 30:tpmd260268. Online ahead of print. doi: 10.4269/ajtmh.26-0268 (PMC13429866; doi:10.4269/ajtmh.26-0268)
Supplement: Supplemental Materials [file tpmd260268.SD1.pdf]

Supplemental Table 1. Breakpoints for disk diffusion testing for Campylobacter.

Antimicrobial Agent	Abbreviation	Resistant (mm)	Intermediate (mm)	Susceptible (mm)	Source
**Quinolone**					
Ciprofloxacin (5 µg)	CIP	≤20	20-24	≥24	a
**Macrolide**					
Erythromycin / Azithromycin (15 µg)	ERY / AZM	≤12	13-15	≥16	a
**Tetracycline**					
Tetracycline (30 µg)	TE	≤22	23-25	≥26	a
**Aminoglycoside**					
Gentamicin (10 µg)	GM	≤12	13-14	≥15	b
**Beta lactam**					
Amoxicillin + Clavulanic Acid (30 µg)	AMC	≤13	14-17	≥18	b
Ampicillin Sulbactam	AMPSUL	≤11	12-14	≥15	b
Imipenem (10 µg)	IMI	≤19	20-22	≥23	b
**Lincosamide**					
Clindamycin (2 µg)	CLI	≤12	13-17	≥18	b
**Other**					
Chloramphenicol (30 µg)	CHL	≤12	13-17	≥18	b
Fosfomycin (200 µg)	FOSF	≤12	13-15	≥16	b
Tigecycline (15 µg)	TIGE	≤14	15-18	≥19	b

a. CLSI. (2016). Methods for Antimicrobial Dilution and Disk Susceptibility Testing of Infrequently Isolated or Fastidious Bacteria. In CLSI guideline M45 (3rd ed.). Clinical and Laboratory Standards Institute.

b. CLSI. (2016). Performance Standards for Antimicrobial Susceptibility Testing. In CLSI supplement M100 (27th ed.). Clinical and Laboratory Standards Institute.
